# Trehalose Accumulation Triggers Autophagy during Plant Desiccation

**DOI:** 10.1371/journal.pgen.1005705

**Published:** 2015-12-03

**Authors:** Brett Williams, Isaac Njaci, Lalehvash Moghaddam, Hao Long, Martin B Dickman, Xiuren Zhang, Sagadevan Mundree

**Affiliations:** 1 Centre for Tropical Crops and Biocommodities, Queensland University of Technology, Brisbane, Queensland, Australia; 2 Department of Plant Pathology and Microbiology, Institute for Plant Genomics and Biotechnology, Texas A&M University, College Station, Texas, United States of America; 3 Department of Biochemistry and Biophysics, Institute for Plant Genomics and Biotechnology, Texas A&M University, College Station, Texas, United States of America; Peking University, CHINA

## Abstract

Global climate change, increasingly erratic weather and a burgeoning global population are significant threats to the sustainability of future crop production. There is an urgent need for the development of robust measures that enable crops to withstand the uncertainty of climate change whilst still producing maximum yields. Resurrection plants possess the unique ability to withstand desiccation for prolonged periods, can be restored upon watering and represent great potential for the development of stress tolerant crops. Here, we describe the remarkable stress characteristics of *Tripogon loliiformis*, an uncharacterised resurrection grass and close relative of the economically important cereals, rice, sorghum, and maize. We show that *T*. *loliiformis* survives extreme environmental stress by implementing autophagy to prevent Programmed Cell Death. Notably, we identified a novel role for trehalose in the regulation of autophagy in *T*.*loliiformis*. Transcriptome, Gas Chromatography Mass Spectrometry, immunoblotting and confocal microscopy analyses directly linked the accumulation of trehalose with the onset of autophagy in dehydrating and desiccated *T*. *loliiformis* shoots. These results were supported *in vitro* with the observation of autophagosomes in trehalose treated *T*. *loliiformis* leaves; autophagosomes were not detected in untreated samples. Presumably, once induced, autophagy promotes desiccation tolerance in *T*.*loliiformis*, by removal of cellular toxins to suppress programmed cell death and the recycling of nutrients to delay the onset of senescence. These findings illustrate how resurrection plants manipulate sugar metabolism to promote desiccation tolerance and may provide candidate genes that are potentially useful for the development of stress tolerant crops.

## Author Summary

Over coming decades, climate change models suggest that droughts and other unpredictable weather patterns will appear more frequently. It is imperative that we develop crops that can survive future climates but continue to yield. Numerous studies have shown that stress tolerance is genetically encoded. Naturally tolerant species therefore represent an ideal starting point for the search for stress tolerance. Resurrection plants belong to a small group of vascular plants that possess unique stress tolerance mechanisms to withstand extreme desiccation with the ability to recover fully upon the availability of water. Here we describe a unique regulatory role for trehalose in the activation of autophagy pathways in *T*.*loliiformis*. We show that *T*. *loliiformis* leaves are alive in desiccated plants and that pre-existing tissues resurrect upon the addition of water. By using a combination of transcriptomics, confocal microscopy and spectroscopy we show that autophagy is induced during dehydration. Notably, we establish that treatment of leaves with trehalose triggers autophagy *in vitro* and that trehalose accumulation in dehydrating leaves correlates with the presence of autophagosomes. We postulate that resurrection plants modulates trehalose metabolism to induce and maintain autophagy pathways that preventing senescence and programmed cell death.

## Introduction

The desiccation tolerant grass, *Tripogon loliiformis*, is a small, tufted diploid grass and member of the poaceae family of cereals that is native to Australia and New Guinea and grows in rocky outcrops and nutrient poor soils with low water retention[1]. In these microenvironments, *T*. *loliiformis* is constantly subjected to environmental extremes and as such has evolved remarkable mechanisms for survival; plants live even after snap-freezing with liquid nitrogen or heating for short periods at temperatures > 60°C[2]. Accordingly, resurrection plants have been investigated for the identification of novel stress tolerance strategies. The advent of “omics” technologies and systems biology approaches provide the experimental power to address the mechanistic details and identify the key mediators of how resurrection plants display the robustness to withstand environmental extremes. Transcriptome, proteome and metabolome studies have been performed on several resurrection plants and have revealed numerous mechanisms that account for the remarkable resilience observed (for a review refer to Dinakar and Bartels, 2013).

Fundamental discoveries of the tolerance strategies utilised by resurrection plants include the early detection of dehydration and shut-down of photosynthesis, the presence of extensive ROS scavenging systems, even in the hydrated state, the accumulation of sugars, as well as the enrichment of transcripts associated with cell wall plasticity[3–12]. Importantly, transcripts and metabolites typically associated with gene profiles observed in seeds are often detected within vegetative tissues, leading to the hypothesis that resurrection plants conform to a dormant “seed-like” state upon drying [3,4,13,14].

The regulation of carbohydrate and nitrogen metabolism also appears to be an integral component of stress tolerance strategies in resurrection plants. In addition to sucrose metabolism, several resurrection plants accumulate substantial levels of the dissarcharide trehalose during drying [9,15,16]. Trehalose is a non-reducing sugar present within a wide-range of organisms including, insects, fungi, bacteria, yeast and several plants and is thought to play a protective role against various environmental stresses[17]. Many of these roles were originally identified in yeast where trehalose plays a protective role by functioning as a chemical chaperone, which prevents protein denaturation, aggregation and influences protein folding through trehalose-protein interactions[17]. In resurrection plants a precise role for trehalose within desiccation tolerance remains elusive as studies have indicated that the trehalose contents accumulated are insufficient to act as either a chaperone or energy source [18]. Treatment with trehalose significantly prolongs the vase life of cut Gladiolus flowers and treated flowers display higher water content and membrane integrity as well as decreased protein degradation. Importantly, a unique role for trehalose from that of other sugars was distinguished by H-NMR spectroscopy which demonstrated that at least in cut flowers trehalose does not play a role in osmotic adjustment but functions to protect vacuolar water [22]. Recently, a new role for trehalose in the induction of mammalian m-TOR independent autophagy pathways was elucidated [19]. Treatment of podocytes with trehalose triggered autophagy and alleviated the effects of mutant proteins associated with Hungtinton and Parkinson disease [19]. Importantly, the effects of trehalose were suppressed by autophagy inhibitors thus linking trehalose with autophagy and not proteasome-mediated pathways [23]. Moreover, trehalose triggered m-TOR independent autophagy did not involve reactive oxygen species and correlated with reduced levels of apoptotic cells[20]. Accordingly, the accumulation of trehalose may play a similar role within the activation of autophagy pathways for the maintenance of tissue vitality in desiccated resurrection plants.

Autophagy is a catabolic cellular process that eukaryotic cells instil as a last ditch effort to restore homeostasis during severe stress and involves the sequestering of cellular material for degradation and recycling or “self-eating”[21]. A double-edged sword, autophagy promotes both survival and death outcomes depending on the context and thus requires intricate regulation to help the cell maintain homeostasis and prevent programmed cell death (PCD) [22,23]. Prolonged stress and excessive autophagy results in “over-eating” of cellular components, insufficient molecular machinery to function and cell death. On the other hand, autophagy is a crucial component of the cellular response system that removes damaged and toxic cellular components, following over stimulation, the accumulation of these components can trigger the induction of PCD pathways[24].

Given the definition of “resurrection” includes “rising from the dead”, the fate of these plants can be viewed in one of two ways, i)desiccated tissues and cells are dead and new growth occurs upon watering or ii) pre-existing tissues arise from a dormant state. The study of cell and tissue fate in resurrection plants provides new insights on how this unique group of plants tolerate extreme environmental challenges; to date, however, little to no progress has been made [25]. Here we show that *Tripogon loliiformis* utilises a myriad of molecular pathways to facilitate the survival of vegetative tissues even in the desiccated state. Key observations include i) “resurrection of pre-existing tissues upon watering, ii) absence of cell death in dehydrated and desiccated tissue, iii) increased detection of autophagy in dehydrating shoots, iv) accumulation of trehalose throughout dehydration, v) trehalose-triggerred induction of autophagy pathways in hydrated leaves and vi) absence of senescence during dehydration and desiccation. These results highlight potential mechanisms that underlie the ability of resurrection plants to survive desiccation and provide functional validation of why some resurrection plants accumulate trehalose at levels that are too low to serve as either osmoprotectants or energy sources. Once activated, autophagy presumably directs the recycling of nutrients and removal of damaged and potentially harmful cellular material to offset senescence and PCD. Effective regulation of autophagy may enable resurrection plants to retain viability even under extreme stress. Due to its broad-sweeping roles from stress-mitigation, anti-aging and prevention of PCD, the study of autophagy in naturally resilient species may provide fundamental information that can be used for the generation of stress-tolerant crops.

## Results

### *Tripogon loliiformis* tissues are revitalised from the desiccated state

**Fig 1:**
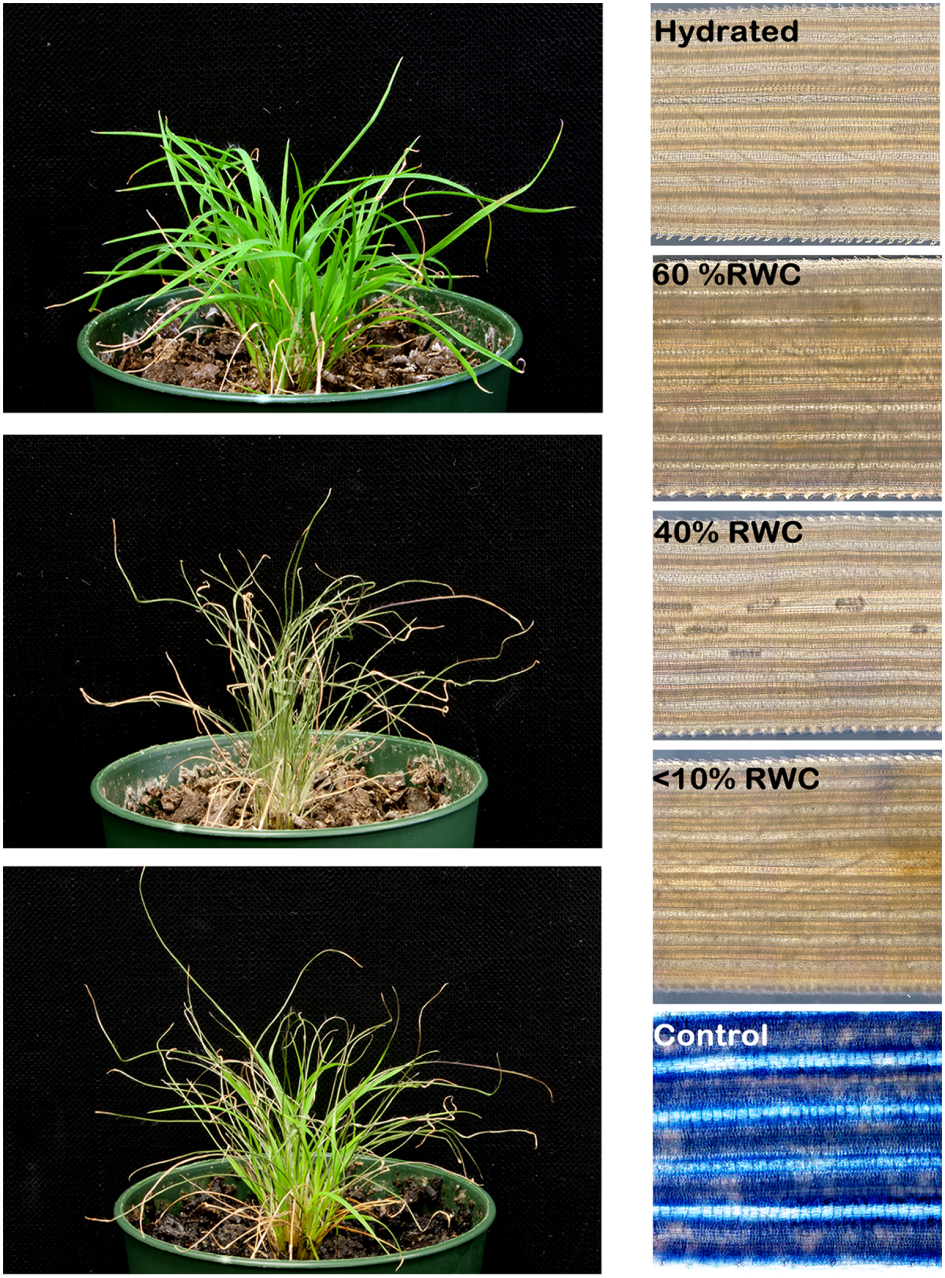
*Tripogon loliiformis* leaf tissues are alive at desiccation. *T*. *loliiformis* plants were grown from seed harvested from a single source and grown in the glasshouse for two months, dehydrated to desiccation and rehydrated with the addition of water. Left Panels—fully hydrated (Top), dehydrating and desiccated (Middle) and 48h after rehydration (Bottom). Right Panels—Leaf tissue was harvested from hydrated and dehydrating plants at 60, 40 and <10% RWC and stained with Evans Blue. As a control, a hydrated leaf was stained after immersion in boiling water for 5 minutes (samples as labelled).

Despite significant research on resurrection plants, little is known of the fate of individual cells throughout desiccation and rehydration. To determine whether pre-existing tissues resurrect or new growth occurs upon watering, time-lapse photography was performed over the rehydration process (S1 Video). The video clearly shows pre-existing tissues resurrecting. The revitalisation of pre-existing growth rather than new growth is an important demarcation and suggests that *T*. *loliiformis*’ vegetative tissues return from a dead-like, cryptobiotic state. To further investigate whether desiccated leaf tissues are alive and “resurrect” upon watering, hydrated, dehydrating (60% and 40% Relative Water Content (RWC)) and desiccated leaves (at <10% RWC) were harvested and stained with Evans blue, an established vital stain that permeates membranes of dead cells. As a control, hydrated leaves boiled for five minutes were also stained. The boiled controls displayed strong Evans blue staining to indicate membrane damage and cell death. As shown in Fig 1, no staining was observed for young leaves at all hydration levels (Fig 1). The absence of Evans blue staining suggests that *T*. *loliiformis* tissues remain viable in the desiccated state.

### *T*. *loliiformis* induces autophagy to suppress senescence and cell death during desiccation

The observation that pre-existing tissues are alive and return from the desiccated state suggests that *T*. *loliiformis* employs efficient cytoprotective measures that prevent cell death during drying. Previous studies suggest that vascular resurrection plants conform to the latter of the two. To test these hypotheses and potentially identify previously unknown stress tolerance strategies we performed RNA-seq analysis on dehydrating (60 and 40% RWC), desiccated (< 10% RWC) and rehydrating *T*. *loliiformis* shoots. As controls, hydrated shoots were also sequenced. These stages of dehydration, desiccation and rehydration were used as they are known to represent vital thresholds that dictate the metabolic activity of resurrection plants. Following Illumina library construction and sequencing, the reads were mapped to a *de novo* assembled blast annotated reference transcriptome. To focus our analysis on whether cellular repair mechanisms were being induced or PCD was being suppressed we compared the expression profile of established plant PCD genes. Key genes associated with senescence were also investigated.

A total of 49 genes associated with apoptototic-like cell death (20), senescence (16) and autophagy (13) were identified in the *T*.*loliiformis* transcriptome and RNA-seq expression analysis revealed the following features, i) transcripts associated with apoptosis and senescence showed less accumulation, ii) transcripts associated with the delay of senescence were increased, and iii) autophagy-related transcripts were more abundant throughout dehydration and desiccation (details for all genes investigated are provided in S1 Table). Subsequent qPCR of select contigs correlated with the fold-changes and the overall trend of the RNA-seq data thus validating the observations (S2 Table).

### *Tripogon loliiformis* suppresses PCD during dehydration and desiccation

**Fig 2:**
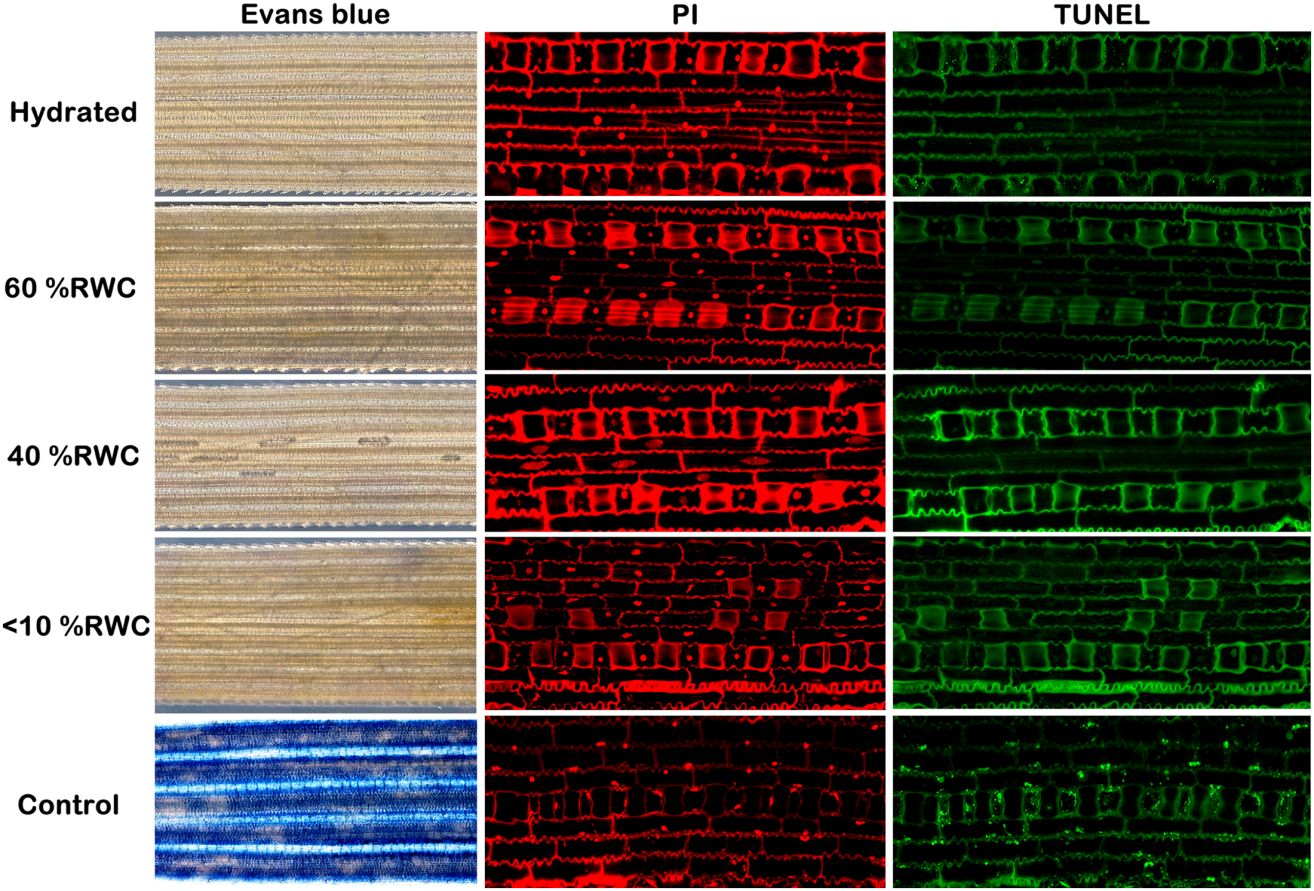
*T*. *loliiformis* suppresses PCD pathway during dehydration and desiccation. Hydrated, dehydrating, desiccated and rehydrated *T*. *loliiformis* leaf tissues were harvested and subjected to TUNEL assay and propidium iodide counter staining for the detection of DNA fragmentation and Apoptotic-like cell death. Hydrated leaves treated with DNase were used as the positive control.

Excessive abiotic and biotic stresses can induce PCD of a few constrained cells presumably for the benefit of the organism as a whole. The transcriptome data shows that during dehydration and desiccation *T*. *loliiformis* suppresses transcription of PCD- and senescence-related genes whilst favouring the expression of autophagy genes. To further investigate whether PCD occurs during dehydration and desiccation of *T*. *loliiformis* leaves, TUNEL assays which allow for the visualisation of DNA fragmentation, an established marker of apoptotic-like cell death, were performed. Consistent with the absence of Evans blue staining and despite numerous TUNEL positive cells in DNase treated controls, no TUNEL positive cells were observed in hydrated, dehydrating or desiccated *T*. *loliiformis* leaves (Fig 2). The Evans blue and TUNEL assay results suggest that *T*. *loliiformis* cells do not succumb to death during desiccation and rather than resurrect, the vegetative tissue is restored from an anhydrobiotic state. The precise mechanisms of how *T*. *loliiformis* cells retain viability in conditions that devastate the majority of angiosperms remain to be elucidated, however, it has been suggested that resurrection plants conform to a dormant seed-like state for survival and longevity upon desiccation.

### Autophagy: A self-eating/cleaning process and facilitator of survival

**Fig 3:**
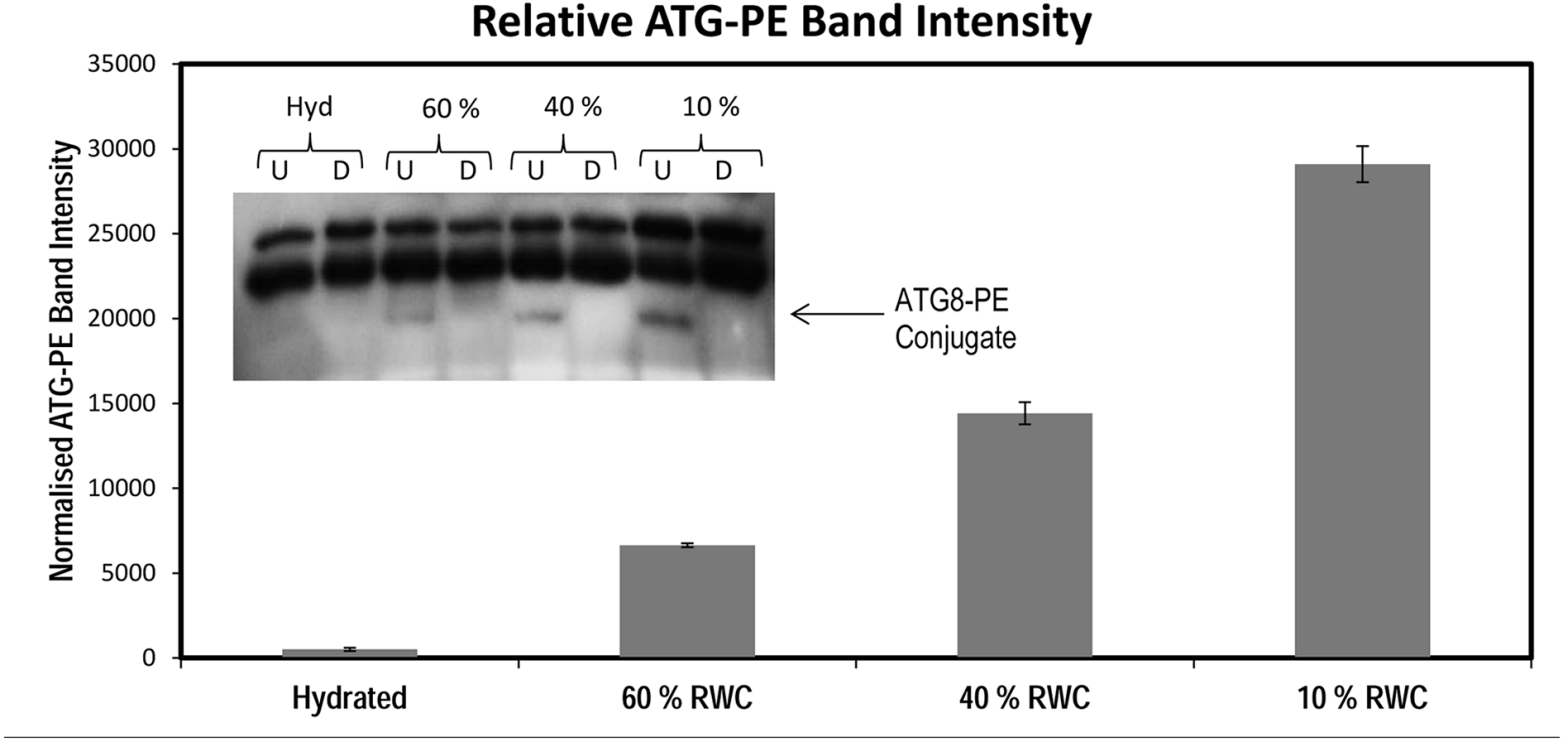
*Tripogon loliiformis* employs autophagy as a pro-survival mechanism during desiccation. Dehydrating (60, 40%RWC) and completely desiccated (<10% RWC) *T*. *loliiformis* leaves were harvested and subjected to Immunoblotting using an ATG8 antibody. ATG8 proteins were made visible by immunoblot analysis with a polyclonal ATG8 antibody following separation by Urea-SDS PAGE*. Approximately 30 μg of total protein as calculated by Bradford assay was loaded for each sample (Ponceau staining of PVDF membrane post-transfer). Protein extracts were subjected to phospholipase D treatment to verify ATG8 lipidation. Signal intensities of the ATG8-PE band were calculated using ImageJ software and were normalised using the Ponceau stained membrane post-transfer for normalisation. Each bar represents the mean ± SEM of triplicate values from a representative experiment. RWC = relative water content, U = undigested, D = digested.

Autophagy pathways have been associated with cellular survival by recycling essential nutrients as well as the removal of damaged organelles and other cellular toxins, thus providing the cells with essential resources for the maintenance of cellular homeostasis. Notably, autophagy is essential for drought and stress tolerance as autophagy deficient plants are significantly more sensitive to drought [24]. The transcriptome data showed that autophagy is triggered in *T*. *loliiformis* during dehydration and desiccation. Could resurrection plants more effectively regulate autophagy pathways than their angiosperm counterparts thus explaining their innate ability to tolerate extreme environments? To further investigate the extent that autophagy pathways are triggered physiologically, hydrated, dehydrating and desiccated *T*. *loliiformis* leaves were i) immunoblotted for detection of transient phosphatidylethanolamine (PE) conjugation of ATG8 and ii) stained with the autophagosome dye Monodansylcadaverine (MDC) and viewed by confocal fluorescence microscopy[26]. The transient conjugation of Atg8 to the amino group of the membrane lipid phosphatidylethanolamine (PE) is an integral component required for autophagosome formation and can be detected by immunoblotting. As shown in Fig 3 and consistent with the transcriptome data, the presence of the ATG8-PE adduct increased during dehydration thus indicating increased accumulation of autophagosomes (Fig 3). Importantly, lipidation of the ATG8 was confirmed by digestion with phospholipase D which converted the ATG8-PE conjugate into the free form (Fig 3)[27]. The immunoblot was subjected to densitometry analysis by ImageJ software after normalisation for equal loading against ponceau stained total proteins transferred to the PVDF membrane, further confirming the accumulation of ATG8-PE throughout dehydration and desiccation (Fig 3 and S1 Fig). MDC stains non-autophagic cells diffusely while autophagosomes are detected as punctate vesicles. Moreover and in accordance with the transcriptome data, no autophagosomes were detected in hydrated *T*. *loliiformis* leaves, multiple autophagosomes however, were observed in dehydrating and desiccated leaves (S2 Fig). Taken together, these results suggest that autophagy is triggered in *T*. *loliiformis* during desiccation.

### A unique role for Trehalose in the survival of resurrection plants

**Fig 4:**
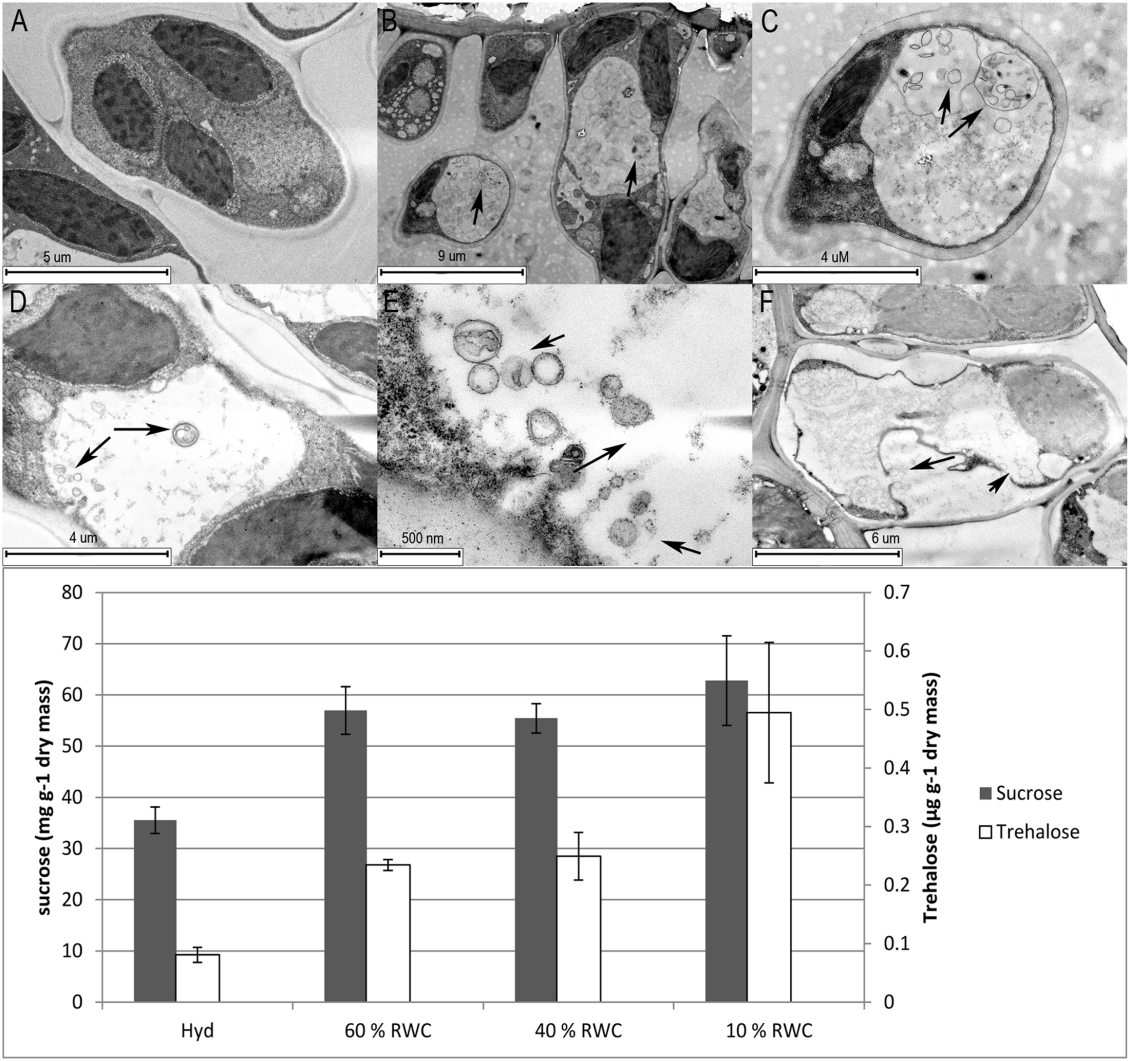
Exogenous application of trehalose triggers autophagy in *T*.*loliiformis*. Hydrated leaves from three month old glasshouse grown *T*. *loliiformis* plants were harvested, divided into 1 cm segments, vacuum infiltrated with 1 and 5 mM trehalose solution containing 1 μM concamycin A prepared in ½ MS basal salts and incubated for 24 hrs. Autophagosomes were visualised by Transmission Electron Microscopy. Top Panel–(A) Concanamycin control, (B) 1 mM trehalose, (C) 1 mM trehalose, higher magnification, (D) 5 mM trehalose, (E) 5 mM trehalose, higher magnification, (F) Tunicamycin control, Bottom panel—The sucrose and trehalose contents of hydrated, dehydrating and desiccated leaves were assessed by GCMS analysis.

The transcriptome, immunoblot and microscopy data suggest that autophagy may play a major role in the preparation of *T*.*loliiformis* in tissues for desiccation and may facilitate the observed prolonged viability compared to sensitive plants. In the search for autophagy regulatory partners we noted that the non-reducing disaccharide trehalose is an inducer of M-TOR independent autophagy pathways in mammals [19,20]. Trehalose has also been reported to accumulate in several resurrection plants [9,28]. It is tempting to speculate that resurrection plants utilise trehalose to trigger autophagy and this represents a unique survival strategy. To further investigate this possibility, GCMS was performed on *T*. *loliiformis* shoots throughout dehydration. Additionally, to confirm that trehalose can trigger autophagy, hydrated *T*. *loliiformis* leaves were treated with trehalose and viewed by Transmission Electron Microscopy (TEM) for the presence of autophagosomes (Fig 4). As shown in Fig 4, GCMS analysis showed that sucrose concentration rose steadily during drying and peaked in desiccated tissue. In accordance with sucrose, trehalose accumulated, albeit at much low levels, throughout dehydration and also peaked in desiccated tissue; hydrated tissues contained low levels of trehalose (Fig 4). These results were consistent with the transcriptome, confocal microscopy and immunoblot data and show that trehalose accumulates in dehydrating and desiccated *T*.*loliiformis* shoots at the same stages of desiccation that autophagosomes are detected.

To further confirm this relationship and assess whether trehalose triggers autophagy directly *in vitro* hydrated *T*.*loliiformis* leaves were treated with 1 or 5 mM trehalose and 1 μM Concanaymcin A solution for 24 hrs and viewed by TEM. As controls, leaves treated with ½ MS salts and Concanamycin A alone were also assessed. Consistent with the previous MDC staining, no autophagosomes were detected in the control samples (Fig 4). Conversely, numerous punctate structures resembling autophagosomes were observed in both the 1 and 5 mM trehalose-treated samples when visualised by TEM (Fig 4). These results provide direct *in vitro* evidence for trehalose-triggered autophagy in plants and suggest that an association exists between trehalose accumulation and the induction of autophagy in *T*.*loliiformis*.

## Discussion

Desiccation tolerance is defined as the ability of a cell or organism to equilibrate water potential with relatively dry air or more specifically to less than 10% cellular relative water content (RWC) at 20°C and a humidity of 50% [29]. Resurrection plants are defined by their ability to withstand desiccation to an air dry state and be revived upon the addition of water. In this study we investigated the underlying mechanisms as to how this occurs. In summary, we uncovered a multi-faceted strategy involving, i) induction of autophagy as a pro-survival mechanism that suppresses PCD, ii) accumulation of trehalose for the induction and maintenance of autophagy during drying, and iii) suppression of PCD and senescence pathways, that is used by *T*. *loliiformis* to withstand desiccation.

Autophagy is a facile yet effective means of increasing longevity during nutrient starvation in heterotrophs and more recently in autotrophs[30]. Studies by Ratnakumar *et al* indicated that autophagy plays important roles within desiccation tolerance in Saccharomyces [31]. Using a combined approach of phenomics, transcriptomics and target gene deletion mutants Ratnakumar *et al* demonstrated that autophagy genes were significantly up-regulated during drying of Saccharomyces cerevisae. Additionally, subsequent gene ontology analysis elucidated that autophagy pathways were significantly enriched during desiccation. The transcriptome and microscopy data suggest that *T*. *loliiformis* not only induces autophagy it triggers the process early during the initial stages of dehydration. Previous studies suggest that resurrection plants are genetically primed to respond to dehydration stress; even in the hydrated state[32]. As part of this priming process and to instigate an optimal cellular environment for the implementation of adaptive measures including the early, induction of autophagy. Resurrection plants respond to water deficit early, closing stomata and shutting down photosynthetic machinery prior to the plant reaching 80% RWC[11]. The closure of stomata and imminent shutdown of photosynthetic machinery during the early stages of dehydration has at least two physiological consequences. It minimises water loss through the stomata thus providing the plant with additional time to implement adaptive strategies. Additionally, the plants are predisposed to caloric starvation due to limited gas exchange and subsequently photosynthesis. It is well established that caloric deficiency and exhaustion of starch stores causes resurrection plants to shift their metabolism to sucrose and amino acid biosynthesis [33]. In addition to shifting metabolism, caloric restriction is known to induce autophagy in both mammal and plant systems [30,34].

**Fig 5:**
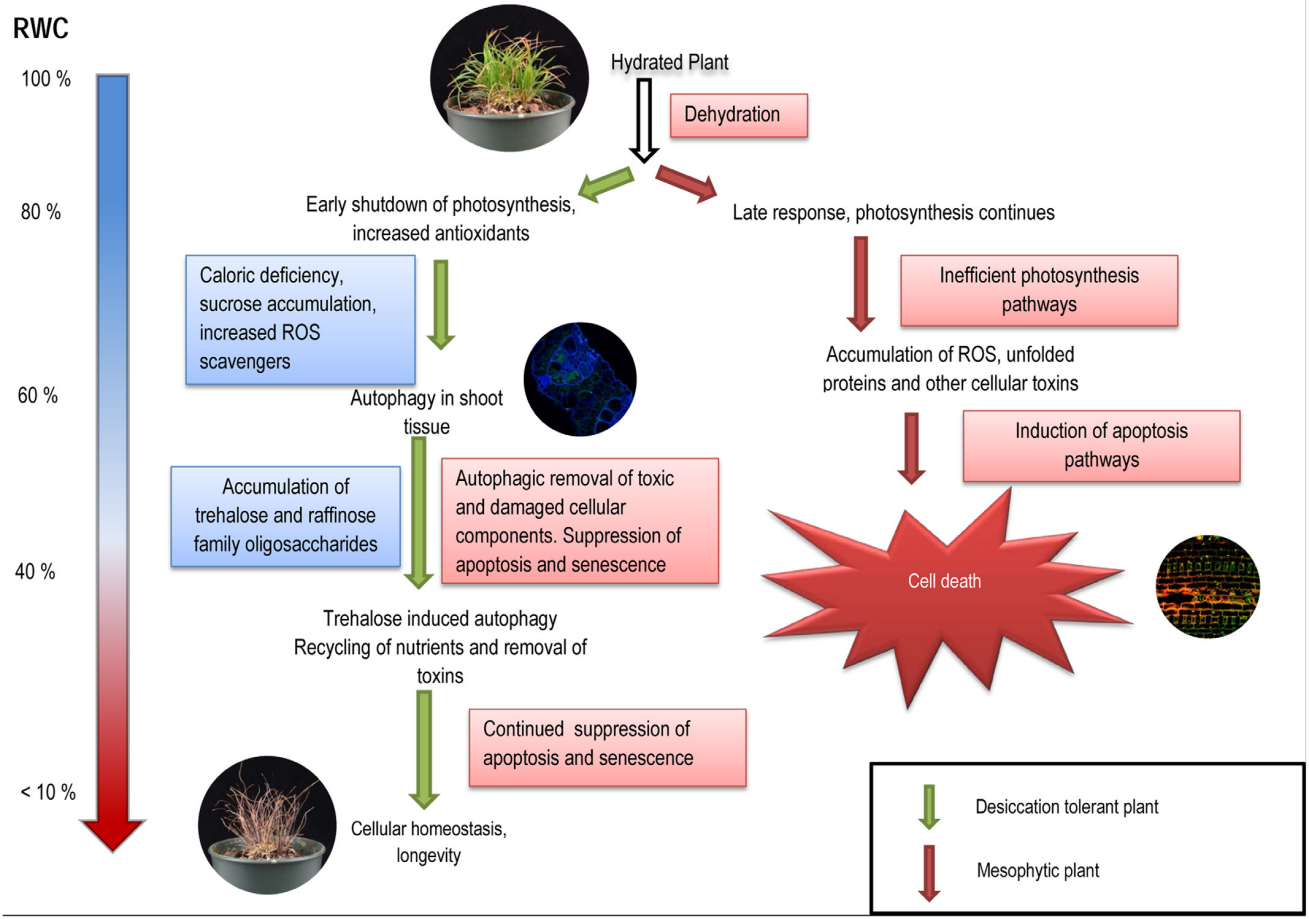
Autophagy-mediated desiccation tolerance in *Tripogon loliiformis*. *Tripogon loliiformis* uses a multi-pronged strategy to survive desiccation stress. I) rapid shutdown of photosynthesis triggers caloric deficiency and the onset of autophagy pathways during the early stages of dehydration. ii) autophagic removal of damaged, unfolded and redundant organelles and proteins as well as ROS mitigates stress to delay the onset of apoptosis. The efficacy of autophagy pathways is bolstered by the accumulation of trehalose throughout dehydration. iii) Nutrient recycling as a product of autophagy mediated breakdown of proteins and organelles suspends “aging” senescence pathways.

Autophagy is associated with extended lifespan in mammals and plants due to its homeostatic role of removing damaged, unwanted cellular components as well as cellular toxins [35,36]. Previous studies have also indicated an antagonistic role of autophagy within apoptosis pathways. In these studies it has been suggested that autophagy suppresses death by removal of apoptotic signals including damaged or misfolded proteins as well as redundant organelles [37–39]. Furthermore, it is suggested that many of the phenotypic phenomena associated with aging are due to decreased induction of autophagy pathways[40]. Studies involving the propagation of Arabidopsis under low-light conditions (similar to the shutdown of photosynthesis) demonstrated autophagic-dependent caloric restriction and longevity [30]. Additionally, Arabidopsis atg9 mutants lacking functional autophagy pathways grown on nutrient deprived media demonstrated premature aging and senescence compared to wild type controls[41]. In the absence of autophagy-directed recycling of cellular contents, nutrients become rapidly limited and senescence ensues, thus, autophagy is involved in the regulation of the aging process. The closing of *T*. *loliiformis* stomata during the early stages of dehydration may play the dual role of conserving water as well as inducing caloric deficiency which triggers autophagy, “cellular cleansing” and prolonged lifespan. A model on how these pathways interact with each other is presented in Fig 5.

Trehalose is a non-reducing disaccharide that is found in bacteria, fungi, insects and plants [42]. A compatible solute, trehalose may decrease cellular osmotic potential to prevent protein denaturation during stresses such as osmotic, temperature and oxidation; trehalose may also act as a chemical chaperone[43]. More recently, trehalose has been implicated within autophagy. Studies by Sakar et al. demonstrated that application of trehalose to human neuroblastoma cells induced autophagy, suppressed apoptosis and protected against Bax-induced cell death[19]. Several resurrection plants are known to accumulate non-reducing sugars as part of their arsenal to survive desiccation[44]. Our GCMS studies indicated that *T*. *loliiformis* accumulates trehalose, peaking in the desiccated state. Given the observation of autophagy at 60, 40 and < 10% RWC and the observation that trehalose triggers autophagy in mammals it is intriguing to speculate on the potential parallels between the roles of trehalose in resurrection plants and mammals. Does trehalose play the dual role of compatible solute and regulator of PCD during dehydration in *T*. *loliiformis* and possibly other resurrection plants? Previous studies have indicated that trehalose suppresses apoptosis in mammals and yeast as well as petal senescence [45–47]. We induced autophagy in hydrated *T*.*loliiformis* leaves with the application of trehalose, thus suggesting that trehalose accumulation may indeed play a role within the regulation of an effective autophagy regime in drying resurrection plants.

Autophagy pathways have been linked with a variety of environmental, nutritional and metabolic stress conditions. A growing body of evidence suggests that autophagy pathways play a dual role promoting both cellular survival and cell death. Pro-survival roles for autophagy include the “controlled sacrifice” of select cellular organelles providing nutritional building blocks to the rest of the cell, thus maintaining energy homeostasis in potentially lethal situations such as nitrogen starvation. Importantly, direct killing by autophagy is yet to be shown conclusively and current hypotheses predict that under certain conditions autophagic digestion of cellular components promotes cell survival rather than death. This however is context dependent, during some circumstances such as the hypersensitive response, autophagy induces death of uninfected bystander cells. In this context, autophagy can be viewed as a survival strategy that is used to avoid excessive cell death by inducing the death of a few select cells for the greater good. The majority of land plants succumb to stress when they drop below 60% RWC, *T*. *loliiformis* and other resurrection plants must ensure that once triggered autophagy pathways are tightly managed to mitigate autophagy-mediated “overindulgence” of cellular contents. Previous studies of resurrection plant dehydration curves including *T*. *loliiformis* indicate that the early closure of stomata allows the plants to retain water for prolonged periods, however, once the water content drops below 60% the plants quickly dehydrate past 40% within a matter of hours[11]. Although speculative, it is possible that the prolonged retention of water at higher RWCs provides vital time for implementation of adaptive strategies however, once the water drops below 60% the plant reaches a threshold of excessive stress which it must pass through rapidly to avoid excessive cellular damage. The rapid descent through this portion of the drying curve may also limit autophagy, thus preventing the switch from pro-survival to cell death or even apoptosis.

### Conclusion

Resurrection plants are unique in their ability to prolong the life of their vegetative tissue in a desiccated state and rejuvenate upon watering. Our results showed that *Tripogon loliiformis* plants do not “resurrect from the dead” but implement pro-survival autophagy pathways, possibly facilitated by the accumulation of trehalose, to prevent apoptosis and senescence during desiccation. Once regarded as a self-destructive process and a form of PCD, autophagy has emerged as a pro-survival process. The induction of autophagy in *T*. *loliiformis* promotes survival by removal of cellular toxins to suppress PCD and the recycling of nutrients to delay onset of senescence. Most importantly, the accumulation of trehalose may maintain the efficiency of autophagy pathways when it is most needed, i.e. 60% RWC and below. The manipulation of autophagy pathways may present great potential for the development of stress tolerant crops.

## Materials and Methods

### Plant materials and dehydration curves

*Tripogon loliiformis* plants were collected from Charleville (Queensland, Australia), transferred to 30 cm pots and allowed to revive in a growth chamber (27°C, 16 hour light period) for three weeks. Five plants were transferred to glasshouse conditions for seed setting. *Tripogon loliiformis* plants were germinated from seeds collected from a single plant and germinated in a growth chamber at 27°C and 16h photoperiod. Fifteen, 65mm pots containing multiple plants were grown for a period of two months. Prior to dehydration all plants were watered to saturation. One day post-watering three replicate samples from the hydrated plants were randomly collected. The remaining plants were dehydrated by withholding water until they were air dry and their relative water content (RWC) dropped below 10%. During dehydration, triplicate samples were collected at 60%, 40% and <10% RWC. Desiccated plants were watered and rehydrated samples collected after 48hrs. The percentage RWC was determined on *T*.*loliiformis* shoots and was calculated according to Barrs and Weatherley, 1962 using the formula (RWC (%) = ((Fresh Weight—Dry Weight)/(Turgid Weight—Dry Weight)) x 100)[48]. All the shoot and root samples were snap frozen in liquid nitrogen and stored at -80°C until RNA extraction.

### Analysis of cell viability by Evans blue staining

To assess cell vitality throughout dehydration Evans blue staining was performed. At least ten shoots from hydrated (≈ 94% RWC), dehydrating (60 & 40% RWC) and desiccated (<10% RWC) *T*. *loliiformis* plants were harvested, placed into 2 mL microfuge tubes and soaked in water for 2 hours to facilitate staining. To serve as a positive control hydrated leaves were boiled for 5 minutes. Following soaking, the water was replaced with 0.25% w/v Evans blue dye and the samples were incubated at room temperature for 20 mins. Stained leaves were rinsed with de-ionised water to remove excess Evans blue dye and stained cells were visually assessed by light microscopy.

### Total RNA extraction and high throughput sequencing

For sequencing analysis, total RNA was isolated from shoot and root tissue of hydrated, dehydrating, dehydrated and rehydrated *T*. *loliiformis* plants using a modified Trizol (Invitrogen) and spin column (Qiagen) method. Details of the extraction protocol can be found in supplemental information. RNA integrity and quality were verified using a Bioanalyser (Agilent technologies). For library preparation, polyadenlyated RNA was enriched, chemically fragmented and cDNA was synthesised using an Illumina RNA-seq kit according to manufacturer’s recommendations. Sequencing of the cDNA libraries was performed at Texas A&M AgriLife Genomics and Bioinformatics service, USA using an Illumina HiSeq 2500 Sequencer (Illumina Inc.). Single-read sequences of length 100 bp were collected. All reads have been deposited in the Sequence Read Archive (SRA) at NCBI, Accession number PRJNA288839.

### RNA-seq analysis

Sequences were assessed for quality control, trimmed to remove the primer and barcode sequences. A readily available de novo assembled, blast annotated *T*. *loliiformis* transcriptome assembly served as a reference for RNA-Seq profiling of the independent cDNA libraries. Over 80% of the reads from each sample were mapped. All data sets were paired and an *in silico* microarray experiment was performed using CLC genomics workbench. Using the Hydrated sample as a reference, each data set was enriched for genes that had a fold change ≥ 2 or ≤ -2, an experimental difference ≥ 5 or ≤ -5 and an adjusted p-value < 0.05 following normalisation by comparison of means.

### Quantitative real-time PCR analysis

Superscript III Reverse Transcriptase (Invitrogen) was used to generate cDNA from 0.8μg of Total RNA using an oligo(dT) (100ρmol) primer. Quantitative PCR was done using a ViiA7 Real-Time PCR System and the SYBR Green PCR Master Mix kit (Applied Biosystems) according to the manufacturer’s instructions using 300 mM primer and 1/100 dilution of cDNA and standard cycling parameters. Gene specific primers for selected genes were designed using Primer3 bioinformatic software (MIT) and are listed in S3 Table. The data analysis was completed using ExpressionSuite Software (Life Technologies). The *Tripogon loliiformis* homologue of Arabidopsis Actin identified from the annotated transcriptome were used for quantitative normalisation. Fold changes were calculated against hydrated tissue.

### Detection of Apoptotic cells by TUNEL assay

To determine whether *T*. *loliiformis* cells were undergoing Apoptotic cell death during desiccation TUNEL assays were performed using the In situ Cell Death Detection Kit, Fluorescein (Roche) as described by Hoang et al., 2014[49]. A hydrated sample was also included as a negative control. TUNEL positive cells were made visible by Nikon A1 Confocal Microscopy.

### Detection of autophagosomes

To detect the presence of autophagosomes in dehydrating *Tripogon loliiformis* plants, shoots from hydrated, dehydrating and desiccated plants (as mentioned above) were excised, immersed in 100 uM Monodansylcadaverine (MDC) and incubated for 30 min at room temperature in the dark [50]. Four leaves for each hydration point were assessed (hydrated, 60%, 40% and 10% RWC). Each leaf was sliced into 1 cm sections and immersed into a 2 mL microfuge tube containing 100 μM MDC stain diluted in ½ MS solution; samples were incubated in the dark for 30 mins. Following staining, each leaf section was washed twice with ½ MS media to remove excess stain and further sectioned by hand using a razor blade. Leaf sections were mounted onto glass slides and observed for the presence of autophagosomes by confocal microscope (Nikon air confocal) using a DAPI filter with excitation and emission at 335nm and 508nm, respectively. Samples were viewed under 40 and 60x oil immersion lenses.

### Trehalose treatment and the detection of autophagosomes by TEM

To assess whether exogenous application of trehalose can trigger autophagy pathways hydrated leaves were treated in triplicate with trehalose and viewed by Transmission Electron Microscopy (TEM). Fully emerged hydrated leaf samples from three month old glasshouse grown *T*. *loliiformis* plants were harvested, divided into 1 cm segments, vacuum infiltrated with a 1 and 5 mM trehalose solution containing 1 μM concamycin A prepared in ½ MS basal salts and incubated for 24 hrs in the light. As a positive control, leaf samples treated with Tunicamycin (5 μg/mL) were also included. For transmission electron microscopy, Trehalose and control treated *T*.*loliiformis* leaf sections were fixed in 3% glutaraldehyde in 0.1M sodium cacodylate buffer followed by post-fixation in 1% osmium tetroxide in 0.1M sodium cacodylate buffer. Samples were subsequently rinsed in UHQ water and dehydrated through a graded series of acetone and embedded in Embed-812 resin. Ultrathin sections were cut on a Leica UC7 ultramicrotome (Leica Microsystems, Wetzlar, Germany) and imaged with a JEOL JEM-1400 transmission electron microscope at an accelerating voltage of 80kV.

### Immunoblot detection of ATG8-PE conjugation

Shoots from dehydrated and desiccated two month old *T*. *loliiformis* plants were snap frozen in liquid nitrogen ground and homogenised in extraction buffer (50mM HEPES-KOH (pH7.5), 150mM KCl, 1mM EDTA, 0.2% Triton-X100, 1mM DTT). Protein samples were quantitated by Bradford assay and approximately 30 μg were separated by Urea-SDS-PAGE (12% Acrylamide, 6M Urea) and transferred onto a polyvinylidene difluoride (PVDF) membrane (Immobilon-PSQ, Millipore membrane). To verify lipidation, protein extracts were digested at 37°C for 1 hour with *Streptomyces chromofuscus* Phospholipase D (Sigma; 250 units mL−1 final concentration) as described by Chung *et al*., 2009)[27]. Immunoblotting analysis was performed using a polyclonal antibody raised against Arabidopsis ATG8 (1:1000 dilution) (ABcam), then visualized using a peroxidase-conjugated goat anti-rabbit IgG (1:1000 dilution) and the Supersignal West Femto Maximum Sensitivity Substrate (Thermo Scientific) according to manufacturer’s instructions. Quantitation of the ATG8-PE band was performed by ImageJ analysis of the Immunoblot normalised against the rubisco protein transferred to the membrane as detected by Ponceau staining. Data are represented as densities of the ATG8-PE bands normalised against their respective Ponceau stained counterparts.

### GC-MS analysis of metabolites in hydrated, dehydrating and desiccated *T*. *loliiformis* shoots

To analyse changes in *T*. *loliiformis* metabolite and potential Trehalose accumulation during dehydration GCMS was performed. Three month old hydrated, dehydrated (60 & 40% RWC) and desiccated <10% RWC plants were harvested, snap-frozen in liquid nitrogen and lyophilised overnight. Following lyphilosation, the dry weight was measured for normalisation and the samples were ground to a powder using a Qiagen tissue lyser (2 x 1 min). Metabolites were extracted and analysed as described by Fiehn *et al* and Hu *et al* [51,52]. Electron ionisation of mass spectra were recorded at a scanning range of 30–650 m/z. For trehalose identification, single ion monitoring with ions 191 and 361 m/z corresponding to the most abundant and specific ions of trehalose methoxyamin 8 trimethylsilyl was performed. All experiments were conducted using three biological replicates.

## Supporting Information

S1 Video *Tripogon loliiformis* resurrects from a dead-like state within 48 hrs. (MOV)

S1 Table *Tripogon loliiformis* supresses cell death and senescence but promotes autophagy during dehydration. (PDF)

S2 Table qRT validation of the RNA_seq transcriptome data. (PDF)

S3 Table Primers used for qRT PCR. (PDF)

S1 Fig Ponceau stain of total protein extracts transferred to PVDF membrane. Total proteins were harvested from dehydrating (60, 40%RWC) and completely desiccated (<10% RWC) *T*. *loliiformis* leaves. Approximately 30 μg of total protein as calculated by Bradford assay was loaded for each sample. Membranes were stained with Ponceau dye (0.5% in 1% Acetic acid) for 10 mins prior to destaining in 1% Acetic acid for 10 mins.
(TIF)

S2 Fig Autophagosomes are observed in dehydrating *Tripogon loliiformis* leaves. Hydrated, dehydrating (60, 40%RWC) and completely desiccated (<10% RWC) *T*. *loliiformis* leaves were harvested and sectioned and treated with the autophagosome specific MDC dye for confocal microscopy. Punctate autophagosome structures were made visible by confocal microscopy using excitation and emission of 335nm and 508nm, respectively.
(TIF)

S1 Text Supplemental Methods. Total RNA extraction and high throughput sequencing.
(PDF)
