# Supplementary material for: Trehalose Accumulation Triggers Autophagy during Plant Desiccation
Source: PLoS Genet. 2015 Dec 3;11(12):e1005705. doi: 10.1371/journal.pgen.1005705 (PMC4669190; doi:10.1371/journal.pgen.1005705)
Supplement: S1 Table — (PDF) [file pgen.1005705.s002.pdf]

Supplemental Table 1: *Tripogon loliiformis* supresses cell death and senescence but promotes autophagy during dehydration

		Shoots			
	Contig#	60%	40%	< 10%	Rehydrated
Cell Death Associated Genes					
Pro-apoptotitic					
Metacaspase family protein	Tl_18779	-2.03	-1.1	-1.06	-1.06
Metacaspase-1-like	Tl_11924	-3.4	-3.11	-2.25	1.33
Metacaspase-4-like	Tl_13100	-9.4	-6.71	-15.67	3.24
Metacaspase-9-like	Tl_53317	55	108	83	0
Apoptosis-related protein	Tl_8053	-1.53	-2.37	-1.9	-1.17
Apoptosis-inducing factor 2-like	Tl_22449	1.31	1.16	-1.35	1.58
E3 ubiquitin-protein ligase AIP2-like	Tl_64322	-4.68	-5	-3.94	3.25
Death-inducer obliterator 1-like	Tl_37912	-4.67	-8.29	-2.64	1.07
Programmed cell death protein 4-like	Tl_10840	8.73	8.52	8.45	1.28
Enhancing RPW8-mediated HR-like cell death 1	Tl_52091	-2.07	-1.86	-1.95	-4.03
PHD finger protein Male Meiocyte death 1-like	Tl_16424	-3.93	-3.11	-3.11	1.82
Programmed cell death 6-interacting protein-like	Tl__6196	1.5	1.6	2.01	-1.01
Aspartic Protease Asp-1	Tl_24898	-26.62	-14.23	-45.89	-9.63
Anti-apoptotic					
Transmembrane BAX inhibitor motif-containing protein 4	Tl_9154	10.67	10.4	9.86	1.57
Putative apoptosis antagonizing transcription factor	T_32992	-13.29	-6.79	-3.48	2.32
Inhibitor of apoptosis-like protein	Tl_44310	3.62	3	1.18	1.55
Inhibitor of Apoptosis (IAP)	Tl_17654	10.33	4.5	1	5.33
Senescence					
Inducers of Senescence					
Protein SRG1-like isoform X1	Tl_33459	-24.9	-38.43	-12.16	-3.7
Senescence-associated protein	Tl_15486	5.71	3.62	5.04	-1.1
Senescence-induced receptor-like serine/threonine-protein kinase	Tl_35516	4.29	1.08	-2.55	1.74
F-box ORE9 (Senescence protein)	Tl_634	-1.43	-2.48	-3.31	-1.98
Leaf senescence protein-like	Tl_6198	-3.01	-3.09	-3.08	-1.31
Senescence-associated protein DH	Tl_8600	-7.24	-2.53	-3.76	-4.56
Senescence-associated protein DIN1-like	Tl_16313	-9.37	-8.52	-9.03	-2.8
Putative senescence-associated protein	Tl_17481	-23.45	-44.17	-263	-1.91
Senescence-associated protein 12	Tl_4022	7.39	7.54	3.33	1.08
Delay of Senescence					
Protein Delay of the onset of senescence	Tl_239	2.03	2.02	-1.16	-2.84

Autophagy					
ATG2	Tl_3592	-1.17	1.12	1.7	-1.51
ATG4	Tl_20336	2.32	2.55	2.39	-1.15
ATG5	Tl_681	2.98	3.15	3.56	1.23
ATG6 (Beclin 1)	Tl_8442	1.44	1.45	1	-1.24
ATG7	Tl_19249	2.28	3.69	3.7	1.3
ATG8F	Tl_33291	1.57	2.39	2.82	-1.59
ATG9	Tl_7270	2.12	2.94	2.85	1.31
ATG10	Tl_10080	-3.26	-4.28	-5.83	-1.2
ATG12	Tl_3955	1.47	1.56	1.58	1.01
ATG13	Tl_24901	1.96	1.59	1.36	-1.56
ATG14	Tl_22742	1.02	-1.05	1.05	-1.12
ATG18G	Tl_17832	2.62	2.86	5.3	-2.18
UPR Genes					
Calreticulin	Tl_3278	-89.2	-109.98	-73.57	-2.93
Calnexin	Tl_1593	-2.31	-4.14	-1.43	1.58
Protein disulfide isomerse (PDI)	Tl_7095	-1.54	-2.17	-2.48	1.16
BIP1	Tl_27222	-2.16	-2.35	1.07	1.06
BiP 2	Tl_2589	-2.06	-3.42	1.71	1.4
BiP3	Tl_13909	-2.34	-3.81	-1.21	1.95
IRE1	Tl_6057	1.44	1.35	1.59	-1.24
