# Supplementary material for: Trehalose Accumulation Triggers Autophagy during Plant Desiccation
Source: PLoS Genet. 2015 Dec 3;11(12):e1005705. doi: 10.1371/journal.pgen.1005705 (PMC4669190; doi:10.1371/journal.pgen.1005705)
Supplement: S2 Table — (PDF) [file pgen.1005705.s003.pdf]

Supplemental Table 2: qRTPCR validation of RNA_seq expression profiles

	RWC	BIP2 (TI_2589)	CRT (TI_3278)	BIP3 (TI_13909)	ATG18 (TI_17832)	Metacaspase Faimly Protein (TI_18779)	ATG7 (TI_19249)	ATG8f (TI_33291)
qRTPCR	60%	-2.39	-57.78	1.00	6.08	1.26	3.50	2.50
	40%	-8.43	-294.05	-5.42	3.49	-1.23	3.46	1.16
	10%	-1.40	-91.52	-1.65	2.60	-1.18	4.16	-1.25
	Reh	1.52	-2.79	2.22	1.68	1.85	1.25	-1.10
RNA_seq	60%	-2.06	-89.2	-2.34	2.62	-2.03	2.28	1.57
	40%	-3.42	-109.98	-3.81	2.86	-1.1	3.69	2.39
	10%	1.71	-73.57	-1.21	5.3	-1.06	3.7	2.82
	Reh	1.4	-2.93	1.95	-2.18	-1.06	1.3	-1.59
