# Supplementary material for: Trehalose Accumulation Triggers Autophagy during Plant Desiccation
Source: PLoS Genet. 2015 Dec 3;11(12):e1005705. doi: 10.1371/journal.pgen.1005705 (PMC4669190; doi:10.1371/journal.pgen.1005705)
Supplement: S3 Table — (PDF) [file pgen.1005705.s004.pdf]

Supplemental Table 3: qRTPCR primer and target sequences

Primer Name	Sequence (5' - 3')	Target
TL_2589F	TGAGGAGCTGAACAACGACC	BIP2
TL_2589R	TCAAGAGCTGCTGGACCTTG	
TL_3278F	GTTTGGCAGGTGAAAGCTGG	CRT
TL_3278R	TCCGTCTTTCAGCTCCTTCG	
TL_13909F	ACTGCAAATAAACCGCCAGC	BIP3
TL_13909R	GAGTGAGGGTGGTGTGGATG	
TL_17832F	CACCAGCAACAACCAGAAGC	ATG18
TL_17832R	TCAAAACGTGATGGCCCAGT	
TL_18779F	ACCATCACCAACTACCGAGC	Metacaspase Faimly Protein
TL_18779R	ATCATCGTCATCCTCGTCGC	
TL_19249F	CCAACTCTTCTCTGCGCTGA	ATG7
TL_19249R	ACAGCTTATCAGTGGCAGCA	
TL_33291F	CTGTCCAACTGTGAGGTCGG	ATG8f
TL_33291R	ATCCCGATCGCATTCCTGTC	
TL_85130R	GCGCGTTGATGATGGAGAAC	
