# Supplementary figures and images for: Trehalose Accumulation Triggers Autophagy during Plant Desiccation
Source: PLoS Genet. 2015 Dec 3;11(12):e1005705. doi: 10.1371/journal.pgen.1005705 (PMC4669190; doi:10.1371/journal.pgen.1005705)

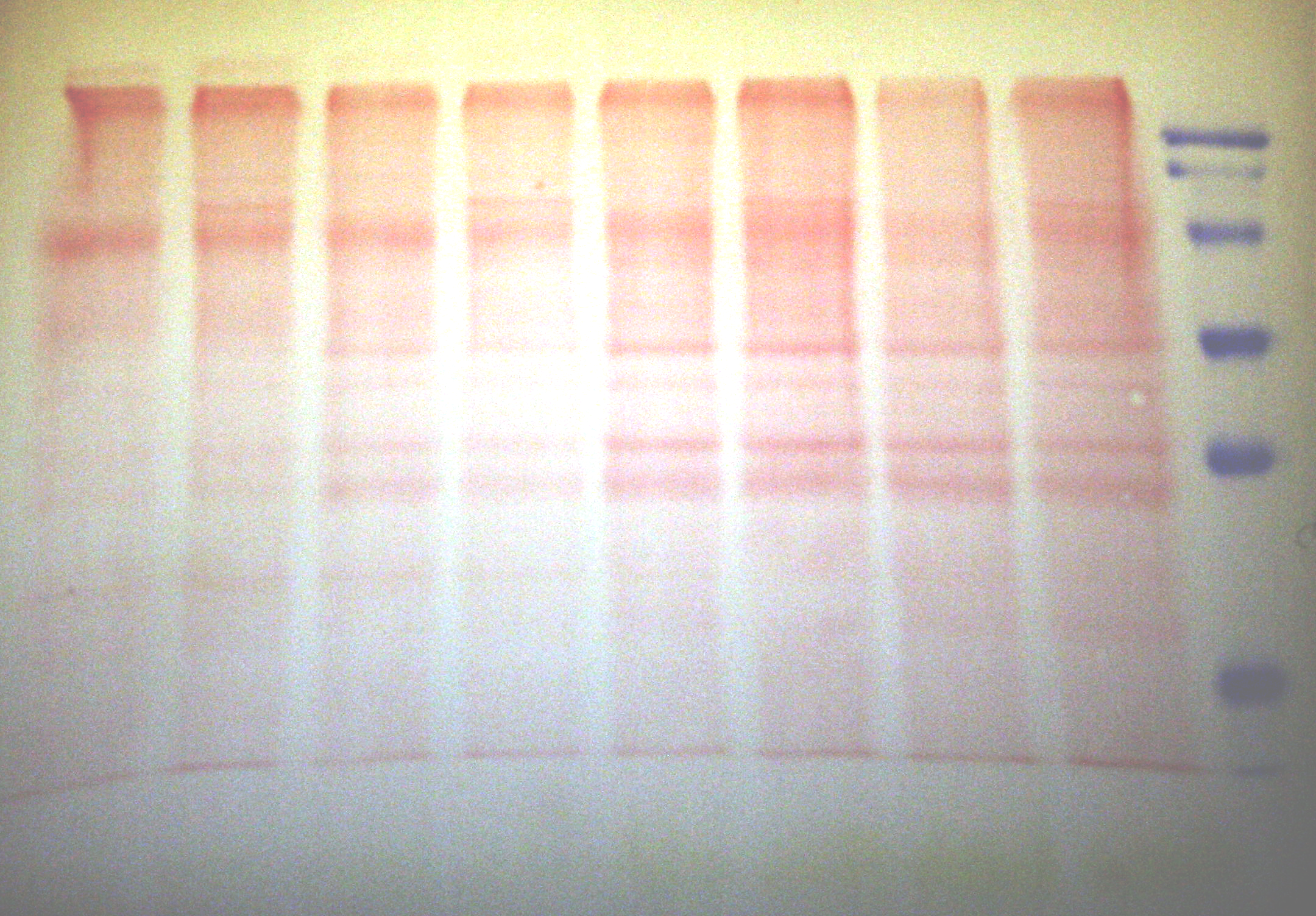

Supplement: S1 Fig — Total proteins were harvested from dehydrating (60, 40%RWC) and completely desiccated (<10% RWC) T. loliiformis leaves. Approximately 30 μg of total protein as calculated by Bradford assay was loaded for each sample. Membranes were stained with Ponceau dye (0.5% in 1% Acetic acid) for 10 mins prior to destaining in 1% Acetic acid for 10 mins. (TIF) [file pgen.1005705.s005.tif]

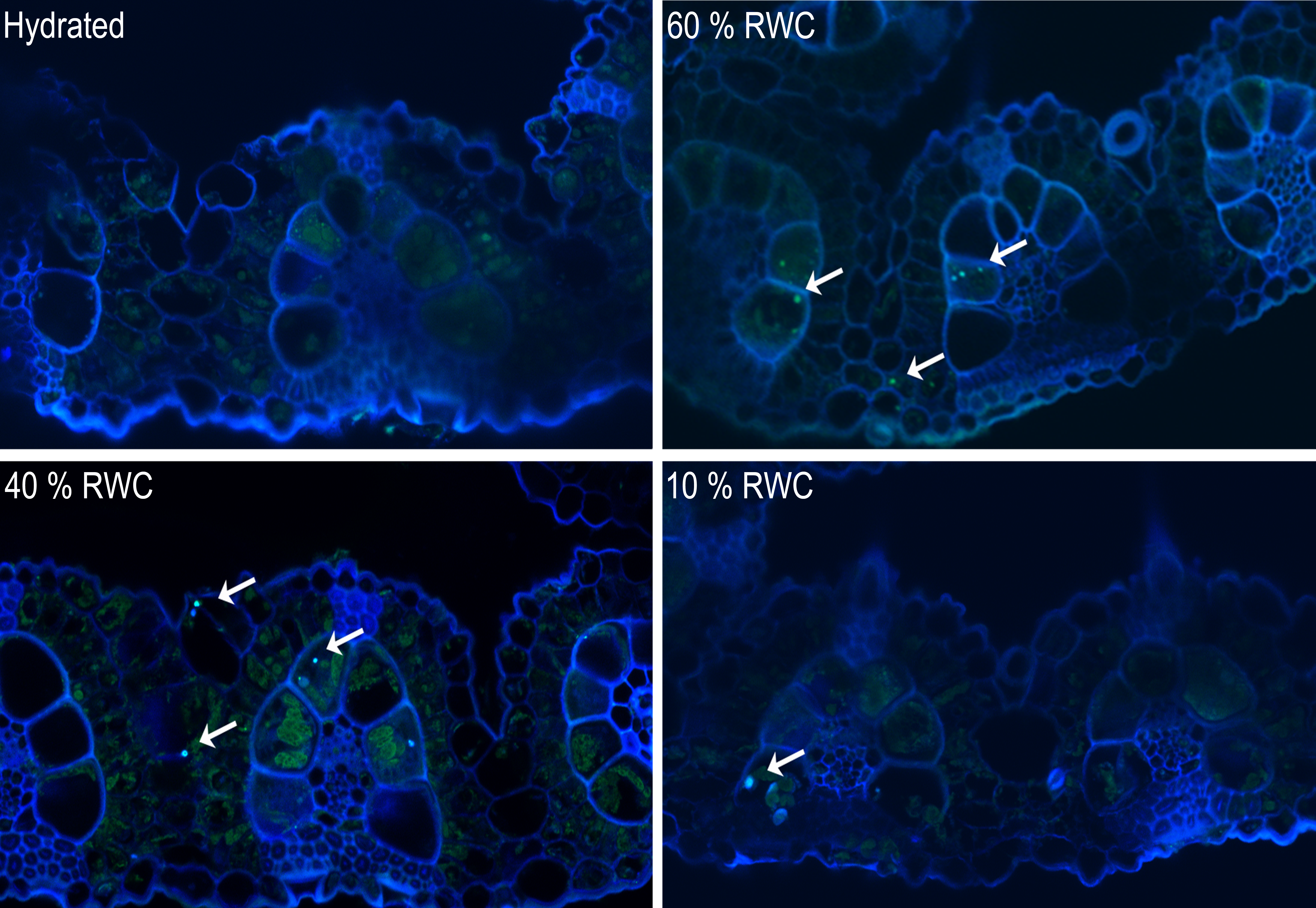

Supplement: S2 Fig — Hydrated, dehydrating (60, 40%RWC) and completely desiccated (<10% RWC) T. loliiformis leaves were harvested and sectioned and treated with the autophagosome specific MDC dye for confocal microscopy. Punctate autophagosome structures were made visible by confocal microscopy using excitation and emission of 335nm and 508nm, respectively. (TIF) [file pgen.1005705.s006.tif]
