# Supplementary material for: Trehalose Accumulation Triggers Autophagy during Plant Desiccation
Source: PLoS Genet. 2015 Dec 3;11(12):e1005705. doi: 10.1371/journal.pgen.1005705 (PMC4669190; doi:10.1371/journal.pgen.1005705)
Supplement: S1 Text — Total RNA extraction and high throughput sequencing. (PDF) [file pgen.1005705.s007.pdf]

Supplemental Methods

Total RNA extraction and high throughput sequencing

Briefly, 100 mg of leaf or root sample was ground to a fine powder using liquid nitrogen and a mortar and pestle. Once ground, samples were transferred to clean microfuge tubes containing 1 mL of Trizol reagent, mixed thoroughly by vortexing and incubated for 5 min at room temperature. Total RNA was clarified by Chloroform extraction. The aqueous phase was transferred to a QIAshredder spin column placed in a 2ml collection tube and centrifuged at maximum speed for 2 min; the flowthrough was retained and transferred to a clean 2 ml collection tube. Approximately 0.5 times 96-100% ethanol was added, mixed by pipetting and the mixture was transferred to a miRNEasy minElute spin column in a 2ml microfuge tube for centrifugation. The remaining wash and elution (30 ul final volume) steps were performed as described in the manufacturer’s instructions. RNA integrity and quality were verified using a Bioanalyser (Agilent technologies). A total of thirty shoot and root sample libraries were prepared.
